# Co-Operative Buying for Hospitals

**Published:** 1914-03-28

**Authors:** 


### CO-OPERATIVE BUYING FOR HOSPITALS.

### A Comparison of Methods in London and New York.

During the last few years the co-operative principle has been put to practical test in London and New York in relation to buying goods for hospital use, but in each case the organisation carrying on the movement has taken the shape of an agency arranging contracts rather than that of a central store or distributing station. The English movement, which never claimed to be anything more than an experiment, has now, temporarily at least, been discontinued, but an American organisation (known as the Hospital Bureau of Standards and Supplies) has apparently reached a stage when it may be regarded as a permanent institution, serving from its head office in New York a number of hospitals in Greater New York and outside.

### The New York Bureau.

The constituent hospitals (which elect the directors) of the Bureau are called "Resident Hospitals," and consist of the institutions joining in the movement situate in the boroughs of Manhattan, Bronx, and Brooklyn of the City of Greater New York. The proportion of the expenses of the undertaking borne by the "Resident Hospitals" varies according to the gross ordinary expenditure of each and the voting power is regulated by the amount of the contributor. There are twelve hospitals of this class.

Other hospitals (numbering twelve) outside Greater New York are allowed to participate and are called "Non-Resident Hospitals." This class, also paying a contribution on the assessment plan, has no voting power under the constitution of the Bureau; as will be seen later, the service of the Bureau is not quite so valuable to the second class as to the first.

The Bureau was formed by Mr. W. V. S. Thorne, and at the end of its fourth year of working had sixty-five agreements in force, as against sixty-three at the close of the previous financial year. The totals include temporary agreements for seasonal goods, such as electric fans in summertime, or short options for single-lot deliveries during the packing season for canned goods and dried fruit.

The executive work is carried out by a committee of seven hospital men, who direct the activities of a purchasing agent. This committee gives considerable attention to the standardisation of goods and the contracts based upon their requirements are arranged by the agent. The advantages obtained by the "Resident Hospitals" are more numerous than those of the other class. In the former instance the possibility of using express deliveries enables the Bureau to supply through its agency the hospitals in Greater New York with perishable articles which, because of difficulty of transport, are not available for those farther afield.

Taking the whole of the present contracts the following may be mentioned as some of the things supplied which would be included under our English heading "Provisions": meat, tea, coffee, cocoa, broths, and soups (it is presumed that the last two articles are tinned goods). Under the heading "Surgery and Dispensary" there is a large range of articles. Dressings of different kinds; rubber goods, including gloves and catheters; and an important list of drugs and chemicals covered by seven agreements. The Bureau also has contracts covering brushes, hardware, paint, blankets, and towels. There are several articles which do not appear in our index of classification, one of which is dictaphones. As might be expected in an American city, ice is found in the list of articles chosen for the operations of the Bureau.

The agreements are described in the Report as local and general, and this suggests that there is not necessarily a single agreement for each article handled, but that, for facilities of delivery, local tradesmen have been selected in some instances. The financial statement shows that the expenditure of the Bureau for the financial year ended September 30 last was 10,415 dollars, of which 8,343 were for salaries and 1,350 for rent; the item "Telephones" cost 196 dollars. The total expenditure was a few dollars more than for the preceding year, and from the fact that the number of contracts was not increased it appears that operations are not at present being extended. It will be noticed that many important articles in common use, such as dairy produce and coal, do not appear upon the list; these perhaps are subjects for future consideration.

### A Centre of Information.

One useful feature of the Bureau is that all members can consult its files and there learn current prices of articles not in the list. The Bureau can also secure for its constituents those trade discounts, usually only given to middlemen, which would not otherwise have been available to consumers. The work of the Bureau has led to much interchange of information regarding practice, quality, and experience; it has, therefore, an educational value which should be considered in addition to the economies secured. As is stated in the Report, "while each institution preserves its autonomy, the use of the standards being optional, the extent to which the members co-operate in supplying information, in using the agreements secured, in advising on questions of practice, measures the advantages to the members and the success of the Bureau. This co-operation is the essence of the Association." We have nothing before us in the way of individual statements from the hospitals concerned, and it would be interesting to examine figures showing cost under certain headings before and during the operations of the Bureau. What may be regarded as general testimony to the efficiency of the movement is evident insomuch as the hospitals attached are presumably satisfied with the results, or they would have withdrawn from the organisation.

The London Experiment.

We now turn to the movement in London during the last few years. As a number of hospitals have worked together in respect of buying goods the movement must be called a co-operative one, although it differs widely in many details of working from other co-operative movements. The keystone of the organisation was the fact that a very large hospital kindly allowed a number of smaller institutions to "come in" at the same price as was paid under its own contracts. The movement continued so long as the predominant partner afforded this assistance, but as soon as the attitude was abandoned the whole thing fell to the ground; it lasted, however, long enough to teach some useful lessons. In the first instance representatives of several hospitals, making a convenient geographical group, all of them north of the river and east of Charing Cross, met together, and after much deliberation and inquiry it was decided, with the help of the large hospital referred to, to enter into contracts for bread and flour, certain drugs and chemicals, and house coal. As at the present time there are practically only three large firms who supply bread to the London hospitals, and as one of them holds by far the greatest number of contracts, very little change was made in the bread supply of the institutions concerned. The price, too, was only affected in the instances of the smaller hospitals, where two or three pence per cwt. was saved. The movement, so far as it concerned coal, was a distinct failure, owing to the very varied requirements of the constituent hospitals; but it was acknowledged that a large proportion of the institutions gained by the co-operative buying of dispensary supplies. As an example, extract of malt was one of the articles chosen in this group, and very large savings were experienced by several hospitals.

When the time came to reconsider the matter some of the hospitals dropped out, not being satisfied that it was worth their while to continue, but some fresh hospitals joined—the geographical area being extended. Bread continued to be bought, coal was discontinued, and the rest of the buying was entirely under the heading, "Surgery and Dispensary," which appears to be here as well as in New York the most suitable field for such enterprise. Certainly in respect of these articles several of the smaller hospitals scored heavily. Of course, one must not be misled by the fact that the times chosen for the commencement of some of the contracts were exceedingly fortunate ones. This was the case one year in respect of cod-liver oil, and it was amusing to see the care with which the supplier checked the quantities delivered during the course of the contract, not letting the hospitals have an ounce over the amount they had specified for.

Naturally, the question of whether such operations were beneficial was much debated in hospital circles, and opinions differed very widely. Some officials gave illustrations of how they were buying more advantageously, forgetting that the dates of the contract to which they referred did not coincide with the dates of the contract under the co-operative scheme. As in the case of many things in our institutional life, the whole of the work of carrying on the organisation was done without any remuneration. A large amount of testing of samples was entailed (this work, by the way, being one of the advantages of the scheme by which all gained), and in addition the clerical work was considerable, including tabular statements of the results of the tenders, preparation of forms of contract, and miscellaneous work of organisation. A large share of the work was done by the authorities of the hospital taking the leading part in the scheme, and by whose co-operation the experiment was made possible.

Comparing the English with the American scheme, it seems clear that if we on this side of the Atlantic are to enter into any further co-operative movement the matter will have to be considered on business lines. Honorary service, except, of course, in respect of committee work, must not play so large a part in the matter. That co-operative buying properly arranged and skilfully worked would in many directions be of benefit to the London hospitals it is difficult to deny. The theory that a large quantity of any article can be bought on more advantageous terms than a small one is unassailable, and the difficulty appears to lie not in purchasing, but in organisation and distribution. Nor is cheapness the only thing to be considered; to mention one possible enterprise amongst many, a co-operative scheme for the purchase of milk could embrace inspection of farms and supply a guarantee of purity it would be difficult to obtain by other means.

